# Retrospective evaluation of a CE-marked AI system, including 1,017,208 mammography screening examinations

**DOI:** 10.1007/s00330-025-11521-4

**Published:** 2025-03-26

**Authors:** Tone Hovda, Marthe Larsen, Marie Burns Bergan, Jonas Gjesvik, Lars A. Akslen, Solveig Hofvind

**Affiliations:** 1 Department of Radiology, Vestre Viken Hospital Trust, Drammen, Norway; 2 Section for Breast Cancer Screening, Cancer Registry of Norway, Norwegian Institute of Public Health, Oslo, Norway; 3 Centre for Cancer Biomarkers CCBIO, Department of Clinical Medicine, Section for Pathology, University of Bergen, Bergen, Norway; 4 Department of Pathology, Haukeland University Hospital, Bergen, Norway; 5 Department of Health and Care Sciences, Faculty of Health Sciences, UiT The Arctic University of Norway, Tromsø, Norway

**Keywords:** Breast cancer, Mammography, Artificial intelligence, Screening

## Abstract

**Objectives:**

To retrospectively evaluate the performance of a CE-marked AI system for identifying breast cancer on screening mammograms. Evidence from large retrospective studies is crucial for planning prospective studies and to further ensure safe implementation.

**Materials and methods:**

We used data from screening examinations performed from 2004 to 2021 at ten breast centers in BreastScreen Norway. In the standard independent double reading setting, each radiologist scored each breast from 1 (negative) to 5 (high probability of cancer). The AI system assigned each examination an NT and an SN score; the NT score aimed to classify examinations as negative with minimal misclassification while the SN score aimed to classify examinations as positive with high confidence. N70 was defined as being among the 70% with the lowest NT score and P3 was defined as being among the 3% with the highest SN score.

**Results:**

A total of 1,017,208 screening examinations were included in the study sample. At N70, 1.8% (107/5977) of the screen-detected and 34.5% (625/1812) of the interval cancers were defined as negative. Using P3 to define cases as positive, 81.5% (4871/5977) of the screen-detected and 19.0% (344/1812) of the interval cancers were defined as positive. Among the screen-detected cancers in N70, 11.2% (12/107) had an interpretation score > 2 by both radiologists.

**Conclusion:**

The AI system performed well according to identifying negative cases and cancer cases. Thus, the AI system can be used to reduce workload for the radiologists and potentially increase the sensitivity of mammography.

**Key Points:**

***Question*** *Results from large mammography screening samples not used in training AI algorithms are important to consider when planning prospective studies and implementation*.

***Findings*** *More than 80% of the screening-detected cancers were classified as positive by AI when considering 3% of the examinations with the highest AI risk score as positive*.

***Clinical relevance*** *A lack of radiologists is a challenge in mammographic screening. Our findings support other studies that suggest the use of AI to reduce screen-reading workload*.

**Graphical Abstract:**

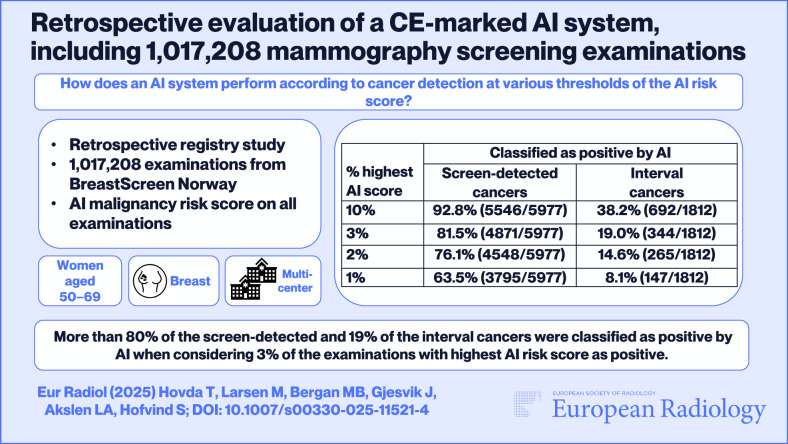

## Introduction

Breast cancer is the most frequent cancer and among the leading causes of cancer deaths among women worldwide [1]. Mammographic screening is recommended as secondary prevention to reduce breast cancer mortality by early detection of the disease [1, 2]. Double reading of screening mammograms is associated with higher sensitivity compared to single reading and should be followed by consensus/arbitration to decide whether to recall the woman [2–4]. Most European countries perform double reading [5].

Mammographic screening has benefits and harms. A false positive screening examination might be considered a burden to the women and is resource-intensive for the breast center, health personnel, and society. Further, a false negative screening examination may lead to delayed diagnosis, either as interval cancer or later-stage screen-detected breast cancer in the consecutive screening round [6]. Lack of breast radiologists is also a challenge to mammographic screening in several European countries [7, 8].

Over the past years, artificial intelligence (AI) has evolved as a promising tool for interpretation in the field of radiology and mammographic screening. Several retrospective and some prospective studies have demonstrated potential for AI in triaging, stand-alone, and as decision support for radiologists in the interpretation of screening mammograms [9–18]. A possible benefit of using AI in the interpretation procedure is a reduction in reading volume without reducing the screening sensitivity. Other benefits may include increased cancer detection and a reduced number of false positive screening results.

Before implementing AI in the interpretation procedure of screening mammograms, thorough evaluation and validation of the algorithms on real screening data is required. The sensitivity of the algorithm at various operating points is needed to ensure an optimal and safe strategy for implementation. Further, knowledge about the associations between AI score and histopathological tumor characteristics and mammographic features is essential for radiologists to ensure safe implementation and long-term cost-effectiveness.

In the current study, we aimed to evaluate the performance of a commercially available AI system for cancer detection in mammography, on retrospective screening data. We used different thresholds of the AI system’s risk scores for classifying negative and positive cases and evaluated the identification of cancer cases at the various thresholds. We also analyzed histopathological tumor characteristics and mammographic features of cancers with different AI scores, as well as the association between AI scores and radiologists’ initial interpretation scores.

## Material and methods

This retrospective study was approved by the Regional Committee for Medical and Health Research Ethics (2018/13294) and had a legal basis in accordance with Articles 6 (1) (e) and 9 (2) (j) of the GDPR. The data was disclosed with a legal basis in the Cancer Registry Regulations Sections 3–1 and the Personal Health Filing System Act section 19a to 19h [19]. The study was based on data from BreastScreen Norway, the national population-based screening program in Norway, administered by the Cancer Registry of Norway, which is a part of the Norwegian Institute of Public Health.

### Study sample

BreastScreen Norway invites all women aged 50–69 to two-view standard digital mammography biennially [20]. During initial screen-reading, the radiologists give each breast an interpretation score between 1 and 5, where 1 indicates negative or benign finding; 2, probably benign; 3, intermediate suspicion; 4, probably malignant; and 5, high suspicion of malignancy.

**Fig. 1:**
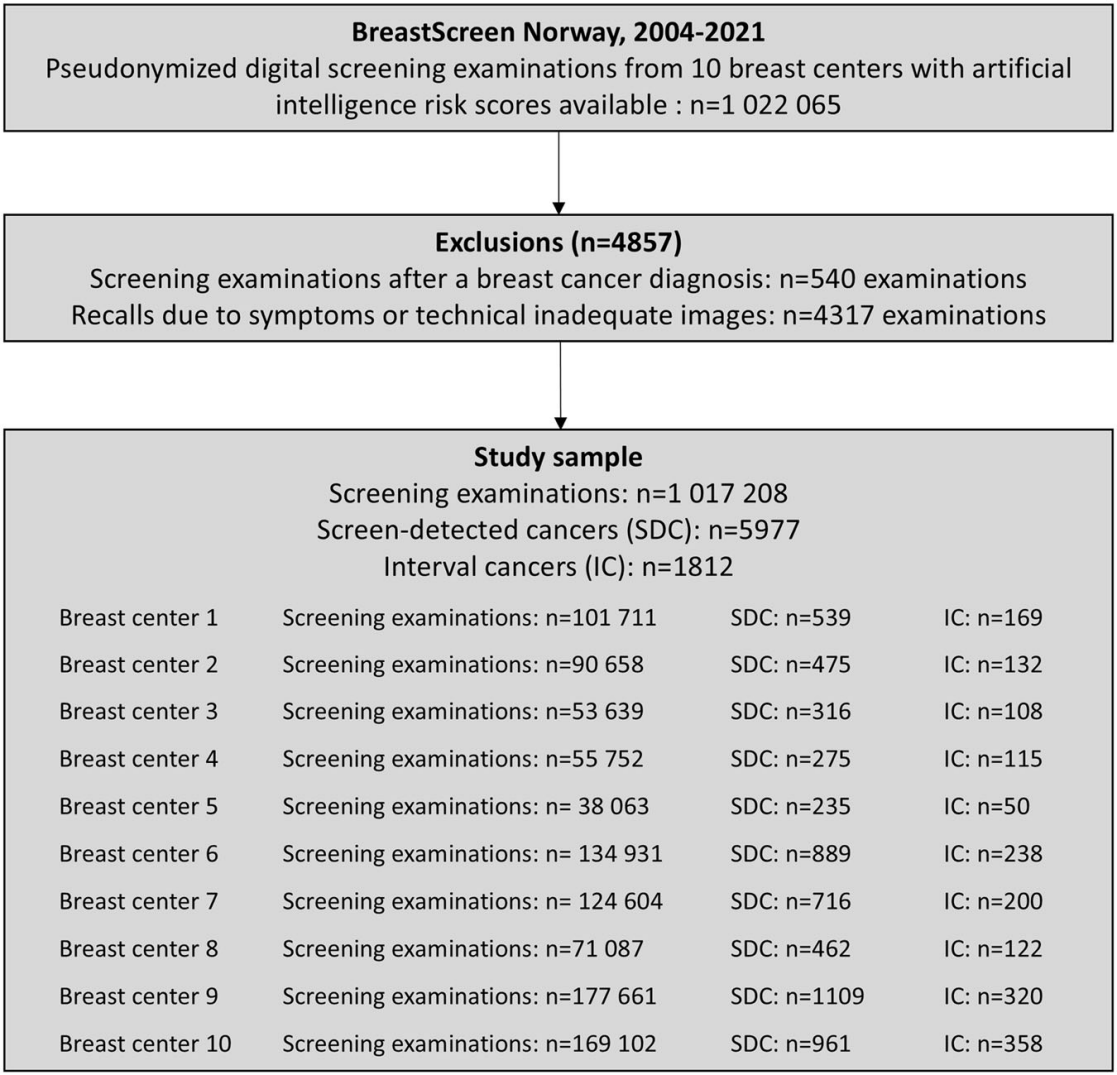
Flow chart of the study sample

In this retrospective observational cohort study, we included standard digital screening mammograms from women screened in BreastScreen Norway, 2004–2021. The AI system could only process examinations with exactly four images and having four images was thus the inclusion criterion for this study. From the overall study sample of 1,022,065 screening examinations with risk scores available from the AI system, we excluded 540 examinations performed after a breast cancer diagnosis and 4317 examinations resulting in a recall due to symptoms or technical inadequate images (Fig. 1). This left 1,017,208 examinations in the final study sample.

### The AI system

All images were analyzed with a commercially available CE-marked and European Medical Device Regulation-certified AI system (Vara version 2.8). For each examination, a “normal triaging” (NT) score and a “safety net” (SN) score were provided by the AI system. Both scores ranged from 0.0 to 1.0, indicating increasing suspicion of breast cancer by increasing score. The AI system is a “two-part system”; the goal of the NT score is to classify examinations as negative with minimal misclassification while the SN score is used to classify examinations as positive with high confidence [21]. The AI system’s NT and SN scores can be configured to best balance detection and workload reduction, e.g., 50% of the examinations can be labeled negative based on the NT score and 1% of the most suspicious examinations can be labeled positive based on the SN score. Examinations not classified as negative or positive by the AI system based on the NT and SN score will be marked as “unclassified” as AI is not confident enough to make a statement about these examinations. These cases should be interpreted by radiologists [21].

### Definition of variables

For the retrospective data, we defined consensus rate as the proportion of cases discussed in a consensus meeting after a score ≥ 2 given by one or both radiologists among all screening examinations during the study period. The recall rate was defined as the proportion of women recalled for further assessment after consensus due to mammographic findings among all screening examinations. Screen-detected cancer was breast cancer (ductal carcinoma in situ (DCIS) or invasive cancer) diagnosed after recall, and interval cancer was breast cancer diagnosed within 24 months after a negative screening examination or 6–24 months after a false positive screening result.

The performance of the NT score was explored for four thresholds. At threshold 1, 90% of the examinations with the lowest NT score were classified as negative (N90), at threshold 2, 80% were classified as negative (N80), at threshold 3, 70% were classified as negative (N70), and at threshold 4, 50% were classified as negative (N50). For the SN score, we explored four different thresholds for selecting positive cases, where P10 classified 10% of the examinations as positive, P3 3%, P2 2%, as positive, and P1 1% as positive.

The radiologists’ interpretation scores were categorized into “1 + 1”, “1 + 2”, “1 + 3/4/5”, “2 + 2”, “2 + 3/4/5”, and “3/4/5 + 3/4/5” based on the highest score for each examination by the two radiologists. Histopathological tumor type was classified as DCIS or invasive cancer. For invasive cancers, we analyzed median tumor diameter (mm), histopathological grade (1–3), lymph node status (positive/negative), and molecular subtypes based on immunohistochemistry. Subtypes were classified according to slightly modified St. Gallen definitions; Luminal A (ER-positive, HER2-negative, Ki-67 ≤ 30% or grade 1 or 2 if Ki-67 is missing), luminal B HER2-negative (ER-positive, HER2-negative, Ki-67 > 30% or grade 3 if Ki-67 is missing), luminal B HER2-positive (ER-positive, HER2-positive), HER2-positive (ER-negative, PR-negative, and HER2-positive), and triple-negative (ER-negative, PR-negative, and HER2-negative) [22]. Mammographic features were described for invasive cancer; mass, spiculated mass, distortion, asymmetry, mass with calcifications or calcifications alone, modified from the BI-RADS classification [23] and in accordance with the classification in the Cancer Registry’s database [20].

### Statistical analysis

We performed descriptive analyses for screen-detected cancer and interval cancer cases based on the different thresholds for selecting negative (N90, N80, N70, and N50) and positive examinations (P10, P3, P2, and P1). Additionally, we calculated the area under the receiver operating characteristics curve (AUROC) for the NT and SN score from the algorithm stratified by breast center and screening equipment, presented with 95% confidence intervals (CI). Cluster bootstrap analysis was performed to account for several attendances for each woman. To descriptively explore associations between AI risk scores (low vs high) and radiologists’ interpretation scores for screen-detected and interval cancers, we stratified results by N70 (low scores), P3 (high scores), and unclassified (not classified as low or high using N70 and P3). The same stratification was used to explore associations between AI risk scores and histopathological characteristics and mammographic features of the tumors. We used Stata (StataCorp. 2023. Stata Statistical Software: Release 18. StataCorp LLC) to analyze the data.

## Results

The study sample included 1,017,208 screening examinations from 10 breast centers in BreastScreen Norway (Fig. 1). All images were full-field digital mammograms, acquired by Siemens MAMMOMAT Inspiration (*n* = 599,358), Philips MDM L50 (*n* = 71,087), Hologic Selenia Dimensions (*n* = 237,105), or GE SenoClaire (*n* = 109,658) (Supplementary Table 1). The smallest center contributed with 38,063 examinations (center 5) and the largest with 177,661 examinations (center 9) (Fig. 1). Mean rate of screen-detected cancer was 5.9 per 1000 examinations (5977/1,017,208; Table 1) and the mean rate of interval cancers was 1.8 per 1000 (1812/1,017,208; Table 1). Using all cancer cases (screen-detected and interval cancers) as the true-status reference value, AUROC for all examinations using the NT score was 0.921 (95% CI: 0.917–0.924) and 0.922 (95% CI: 0.918–0.925) using the SN score (Table 2). Using the NT score, the AUCROC for examinations from the breast centers using Siemens equipment was 0.927 (95% CI: 0.923–0.932), 0.900 (95% CI: 0.884–0.916) for the breast center using Philips equipment, 0.921 (95% CI: 0.914–0.929) for the examinations performed with Hologic and 0.895 (95% CI: 0.882–0.907) for the examinations performed with GE (Supplementary Table 1).

**Table 1:** Consensus, recall, screen-detected cancers (SDC), and interval cancers (IC) for the different breast centers in the study sample

Breast center	Consensus, *n* (%)	Recall, *n* (%)	SDC, *n* (*n* per 1000)	IC, *n* (*n* per 1000)
Breast center 1	5505 (5.4%)	2485 (2.4%)	539 (5.3 per 1000)	169 (1.7 per 1000)
Breast center 2	6626 (7.3%)	2589 (2.9%)	475 (5.2 per 1000)	132 (1.5 per 1000)
Breast center 3	4685 (8.7%)	1788 (3.3%)	316 (5.9 per 1000)	108 (2.0 per 1000)
Breast center 4	2549 (4.6%)	1043 (1.9%)	275 (4.9 per 1000)	115 (2.1 per 1000)
Breast center 5	3467 (9.1%)	1097 (2.9%)	235 (6.2 per 1000)	50 (1.3 per 1000)
Breast center 6	12,833 (9.5%)	5098 (3.8%)	889 (6.6 per 1000)	238 (1.8 per 1000)
Breast center 7	13,769 (11.1%)	4013 (3.2%)	716 (5.7 per 1000)	200 (1.6 per 1000)
Breast center 8	5561 (7.8%)	2969 (4.2%)	462 (6.5 per 1000)	122 (1.7 per 1000)
Breast center 9	12,907 (7.3%)	5397 (3.0%)	1109 (6.2 per 1000)	320 (1.8 per 1000)
Breast center 10	21,395 (12.7%)	6361 (3.8%)	961 (5.7 per 1000)	358 (2.1 per 1000)
Total	89,297 (8.9%)	32,840 (3.2%)	5977 (5.9 per 1000)	1812 (1.8 per 1000)

Rates (% and per 1000) were calculated using the number of examinations as the denominator

**Table 2:** Area under the receiver operating curve (AUROC) + 95% CIs for each breast center estimated using the NT score and SN score by the AI system

	NT score		SN score	
	AUROC_SDC (95% CI)	AUROC_SDC + IC (95% CI)	AUROC_SDC (95% CI)	AUROC_IC + SDC (95% CI)
Breast center 1	0.980 (0.974–0.986)	0.924 (0.911–0.937)	0.980 (0.974–0.986)	0.927 (0.914–0.939)
Breast center 2	0.983 (0.977–0.988)	0.937 (0.925–0.949)	0.982 (0.977–0.988)	0.938 (0.926–0.950)
Breast center 3	0.971 (0.963–0.980)	0.913 (0.896–0.930)	0.971 (0.963–0.979)	0.913 (0.896–0.930)
Breast center 4	0.982 (0.976–0.988)	0.922 (0.906–0.939)	0.982 (0.976–0.988)	0.924 (0.908–0.940)
Breast center 5	0.977 (0.968–0.986)	0.932 (0.912–0.951)	0.980 (0.969–0.986)	0.933 (0.914–0.952)
Breast center 6	0.978 (0.975–0.982)	0.930 (0.922–0.939)	0.979 (0.975–0.983)	0.931 (0.923–0.940)
Breast center 7	0.975 (0.970–0.980)	0.927 (0.917–0.938)	0.975 (0.970–0.980)	0.929 (0.919–0.939)
Breast center 8	0.961 (0.952–0.969)	0.900 (0.884–0.916)	0.959 (0.951–0.968)	0.883 (0.870–0.895)
Breast center 9	0.977 (0.973–0.981)	0.927 (0.919–0.935)	0.977 (0.973–0.981)	0.929 (0.920–0.937)
Breast center 10	0.962 (0.956–0.968)	0.896 (0.886–0.907)	0.961 (0.956–0.967)	0.893 (0.883–0.903)
Total	0.974 (0.972–0.976)	0.921 (0.917–0.924)	0.974 (0.972–0.976)	0.922 (0.918–0.925)

AUCROC was estimated using only screen-detected cancers (AUROC_SDC) and using all cancers (interval cancers and screen-detected cancers as true reference status, AUROC_IC + SDC) in the sample

### NT and SN

When defining 90% of the examinations as negative based on the NT score (N90), 7.1% (423/5977) of the screen-detected cancers were classified as negative by AI (Table 3a). At N70 and N50, 1.8% (107/5977) and 0.4% (21/5977) of the screen-detected cancers were classified as negative, respectively. For interval cancers, 61.6% (1116/1812) were classified as negative at N90, 34.5% (625/1812) at N70, and 18.9% (342/1812) at N50. Using all cancer cases (screen-detected and interval cancers), 9.4% (732/7789) were classified as negative at N70.

**Table 3:** Screen detected cancers (SDC), interval cancers (IC), and all cancer cases (SDC + IC) classified as negative/positive using the (a) normal triaging score and (b) the safety net score

(a) Normal triaging			
	SDC classified as negative by AI	IC classified as negative by AI	SDC + IC classified as negative by AI
N90	7.1% (423/5977)	61.6% (1116/1812)	19.8% (1539/7789)
N80	3.4% (200/5977)	46.3% (838/1812)	13.3% (1038/7789)
N70	1.8% (107/5977)	34.5% (625/1812)	9.4% (732/7789)
N50	0.4% (21/5977)	18.9% (342/1812)	4.7% (363/7789)

(b) Safety net			
	SDC classified as positive by AI	IC classified as positive by AI	SDC + IC classified as positive by AI
P10	92.8% (5546/5977)	38.2% (692/1812)	80.1% (6238/7789)
P3	81.5% (4871/5977)	19.0% (344/1812)	67.0% (5215/7789)
P2	76.1% (4548/5977)	14.6% (265/1812)	61.8% (4813/7789)
P1	63.5% (3795/5977)	8.1% (147/1812)	50.6% (3942/7789)

In N90, N80, N70, and N50, 90%, 80%, 70%, and 50% of the examinations with the lowest normal triaging score by the artificial intelligence system were defined to be negative. In P10, P3, P2, and P1, 10%, 3%, 2%, and 1% of the examinations with the highest safety net score were defined to be positive

If all interval cancers were considered as false negatives, the sensitivity of standard independent double reading in the study sample was 76.7% (5977/7789). In a scenario where AI was used to identify negative cases and reduce the screening volume by 70% (no radiologists involved in the screen-reading of examinations in N70), sensitivity was estimated to be 75.3% (5977 − 107)/7789.

Using the SN score to define examinations as positive, 92.8% (5546/5977) of the screen-detected cancers were classified as positive by AI at P10, 81.5% (4871/5977) with P3, and 63.5% (3795/5977) with P1 (Table 3b). For interval cancers, 38.2% (692/1812) were classified as positive at P10, 19.0% (344/1812) at P3, and 8.1% (147/1812) at P1.

Among the 21 screen-detected cancers classified as negative at N50, 107 were classified as negative at N70, 200 as negative at N80, and none were classified as positive using the SN (P10, P3, P2, or P1). A total of 15 of the 423 screen-detected cancers (3.5%) classified as negative at N90 were classified as positive at P10. At P1, P2, and P3, none of these cancer cases were identified by the SN. For interval cancers, 10 of the 1116 cases (0.9%) classified as negative at N90 were classified as positive at P10.

If all positive cases selected at P1 were to be discussed in the consensus meeting in addition to cases already selected for consensus by radiologists, the consensus rate in this sample would increase from 8.8% (actual rate in the screening setting without AI) to 9.2%. Among the 147 interval cancers in P1, 51 were already selected for consensus by one or both radiologists, and 21 of these cases had a recall assessment that turned out negative. A total of 96 interval cancers in P1 were selected by AI only.

### The radiologists’ interpretation scores of cancer cases in N70, unclassified, and P3

**Fig. 2:**
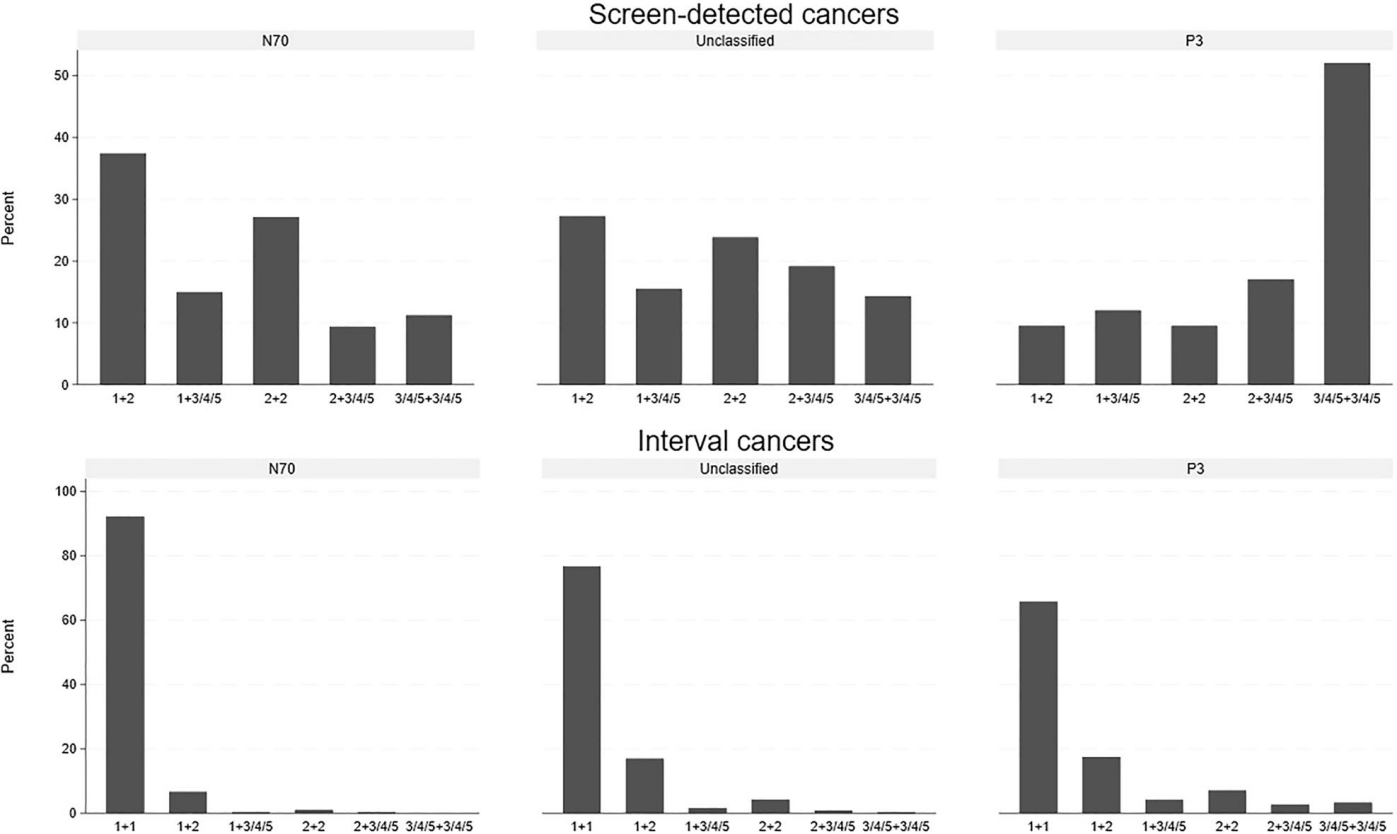
Interpretation scores by the radiologists for screen-detected and interval cancer cases among the 70% with the lowest NT score (N70), 3% with the lowest SN score (P3), and unclassified (not in N70 or P3)

Among the screen-detected cancers defined as negative using N70, 37.4% (40/107) had interpretation scores of 1 by one radiologist and 2 by the other, and 11.2% (12/107) had an interpretation score of 3 or higher by both radiologists (Fig. 2 and Supplementary Table 2). A total of 65.7% (226/344) of the interval cancers defined as positive at P3 were scored negative, 1, by both radiologists. At P3, most of the interval cancers with interpretation score 1 + 2, 86.7% (52/60), were not recalled for further assessment in the regular screening setting.

### Histopathological tumor characteristics and mammographic features of invasive cancer cases in N70, unclassified, and P3

The percentage of invasive cancers was 85.1% (91/107) in N70, 79.2% (791/999) in unclassified, and 83.4% (4062/4871) in P3 (Table 4). Median tumor diameter for invasive screen-detected cancers in N70 was 10 mm (IQR: 8–15), 10 mm (IQR: 7–15) in unclassified, and 13 mm (IQR: 9–18) in P3. A total of 16.9% (15/89) were histologic grade 3 in N70, 19.9% (155/778) in unclassified, and 20.4% (824/4035) in P3. For luminal A, the percentages for N70, unclassified, and P3 were 76.3% (58/76), 73.4% (505/688), and 70.9% (2546/3593), respectively. The most common mammographic features for screen-detected cases in N70 were asymmetry (34.9%, 29/83) and mass (31.3%, 26/83). In P3, the most common feature was spiculated mass (46.8%, 1776/3796).

**Table 4:** Histopathological tumor characteristics and mammographic features of invasive screen-detected and interval cancers stratified by N70, unclassified, and P3, where N70 is the 70% with the lowest NT score by the AI system, P3 is the 3% with the highest SN score, and unclassified are cases not classified as negative (N70) or positive (P3)

	Screen-detected cancers, *n* = 5977			Interval cancers, *n* = 1812		
	N70, *n* = 107	Unclassified, *n* = 999	P3, *n* = 4871	N70, *n* = 625	Unclassified, *n* = 843	P3, *n* = 344
Invasive cancers, *n* (%)	91 (85.1%)	791 (79.2%)	4062 (83.4%)	582 (93.1%)	795 (94.3%)	320 (93.0%)
Histopathological characteristics of invasive cancers						
Tumor diameter^*^, mm median (IQR)	10 (8–15)	10 (7–15)	13 (9–18)	16 (11–23)	18 (13–25)	17 (12–25)
Histologic grade, *n* (%)						
Grade 1	29 (32.6%)	247 (31.8%)	1113 (27.6%)	67 (11.9%)	104 (13.4%)	53 (17.0%)
Grade 2	45 (50.6%)	376 (48.3%)	2098 (52.0%)	278 (49.2%)	366 (47.0%)	179 (57.6%)
Grade 3	15 (16.9%)	155 (19.9%)	824 (20.4%)	220 (38.9%)	308 (39.6%)	79 (25.4%)
Data not available	2	13	27	17	17	9
Lymph node involvement, *n* (%)	16 (18.4%)	94 (12.1%)	854 (21.4%)	171 (30.9%)	265 (35.3%)	102 (34.5%)
Data not available	4	24	67	29	45	24
Immunohistochemical subtypes, *n* (%)						
Luminal A	58 (76.3%)	505 (73.4%)	2546 (70.9%)	254 (48.8%)	330 (47.4%)	165 (60.2%)
Luminal B HER2-negative	5 (6.6%)	63 (9.2%)	499 (13.9%)	89 (17.1%)	155 (22.2%)	51 (18.6%)
Luminal B HER2-positive	4 (5.3%)	41 (6.0%)	241 (6.7%)	40 (7.7%)	62 (8.9%)	20 (7.3%)
HER2-positive	2 (2.6%)	19 (2.8%)	124 (3.5%)	40 (7.7%)	59 (8.5%)	18 (6.6%)
Triple negative	7 (9.2%)	60 (8.7%)	183 (5.1%)	98 (18.8%)	91 (13.1%)	20 (7.3%)
Data not available	15	103	469	61	98	46
Mammographic features of invasive cancers						
Mass	26 (31.3%)	214 (29.8%)	579 (15.3%)	33 (14.5%)	58 (16.6%)	12 (8.5%)
Spiculated mass	18 (21.7%)	227 (31.6%)	1776 (46.8%)	108 (47.6%)	152 (43.4%)	60 (42.3%)
Distortion	7 (8.4%)	28 (3.9%)	125 (3.3%)	12 (5.3%)	24 (6.9%)	6 (4.2%)
Asymmetry	29 (34.9%)	166 (23.1%)	514 (13.5%)	55 (24.2%)	73 (20.9%)	32 (22.5%)
Mass with calcifications	2 (2.4%)	22 (3.1%)	349 (9.2%)	16 (7.1%)	26 (7.4%)	13 (9.2%)
Calcifications alone	1 (1.2%)	62 (8.6%)	453 (11.9%)	3 (1.3%)	18 (4.9%)	19 (13.4%)
Data not available	8	72	266	355	445	178

^*^A total of 674 invasive screen-detected cancers and 458 invasive interval cancers among women who received neoadjuvant treatment/therapy were excluded

For interval cancers, the percentage of invasive cancers was 93.1% (582/625) in N70, 94.3% (795/843) in unclassified, and 93.0% (320/344) in P3 (Table 4). A total of 38.9% (220/565) were histologic grade 3 in N70, 39.6% (308/778) in unclassified, and 25.4% (79/311) in P3. A total of 18.8% (98/521) was triple negative in N70, 13.1% (91/697) in unclassified, and 7.3% (20/274) in P3. At diagnosis, the most common mammographic feature for interval cancer cases in N70 was spiculated mass (47.6%, 108/227). In unclassified and P3, 43.4% (152/350) and 42.3% (60/142) of the cases were spiculated masses, respectively. Calcifications alone were registered for 1.3% (3/227) in N70, 4.9% (18/350) in unclassified, and 13.4% (19/142) in P3.

## Discussion

In this study using retrospective screening data merged with results of an AI system for more than 1 million screening examinations performed at ten breast centers in BreastScreen Norway, we found that 1.8% of the screen-detected cancers were classified as negative by AI when defining 70% with the lowest AI risk score (NT score) as negative. Using the SN score to classify the top 3% as positive, 81.5% of the screen-detected cancers were classified as positive by AI.

In a study using a nearly identical sample of Siemens examinations, comparable AUROC estimates were reported by another CE-marked AI system when including screen-detected cancers only (0.98 in this study vs 0.97 for the other AI system) and also interval cancers + screen-detected cancers (0.93 in this study vs 0.93 for the other AI system) as true reference status [15]. In the same study, 92% of screen-detected and 45% of interval cancers were among the top 10% with the highest AI risk score. In this study, 93% of the screen-detected and 38% of the interval cancers were identified at P10.

Using the SN score, we found 8.1% of the interval cancers among the top 1% with the highest AI score (P1) at the screening examinations prior to diagnosis. We did not include information about the location of AI markings and true cancer location in our analysis, but a recent study using the same AI system reported that 91.2% (103/113) of false negatives identified at an operating point of 99% specificity (exemplary operating point to emulate a recall rate of approximately 1%) had correct location [9]. This is a promising result regarding earlier detection of interval cancers with AI support in screen-reading. On the other hand, in the total sample of 2396 interval cancers, the percentage of false negatives with the correct location identified was 4.3% [9]. The operating point(s) for identifying suspicious cases must be considered and decided carefully to balance potential benefits and harms, i.e., increased sensitivity and reduced workload for the radiologists vs higher consensus and/or recall rate.

We found that 12 (11.2%) of the screen-detected cancers classified as negative at N70 had an interpretation score of 3, 4, or 5 by both radiologists. Despite a limited number of cases in this study sample, the AI system should most likely have assigned these cases a higher score. For interval cancers with an interpretation score of 2 or higher by one or both radiologists at screening prior to diagnosis, a recent study demonstrated that 21% were classified as missed in an informed review, but only a few of these were recalled [24]. Thus, using AI as decision support might reduce the number of missed cancers by adding information to the radiologists’ decision-making process.

For screen-detected cancers, results on tumor diameter, histologic grade, lymph node involvement, and subtypes indicated prognostically favorable characteristics of screen-detected cancer with low vs high AI scores. For interval cancers, the opposite was observed. Interval cancers in N70 had less prognostic favorable tumor characteristics with higher proportions of histological grade 3 and triple negative cancers than interval cancers in P3, indicating that cancers not detected by radiologists or AI at screening are fast-growing. This is in keeping with other studies from BreastScreen Norway, retrospectively analyzing other algorithms [14, 15]. In contrast to P3, screen-detected cancers detected by radiologists but not AI (N70) were more frequently classified as masses and asymmetries than spiculated masses. Masses and asymmetries often represent benign findings, and radiologists’ access to prior mammograms is important in the interpretation process. For interval cancers, the mammographic features were registered at diagnosis, not at screening, and despite a few cases, the proportions for calcifications (13.4% at P3 vs 1.3% at N70) might indicate a potential for earlier diagnosis with AI in screen-reading for calcifications.

The large number of examinations and mammograms from different equipment vendors and from all four Norwegian regional health authorities was a strength to our study. Limitations were related to the retrospective approach which limits the generalizability of results to a prospective screening setting. Another limitation was that the AI system provided an overall AI risk score for each examination, not for each breast, with no information on whether the finding considered suspicious by AI corresponded to the actual cancer location.

To conclude, the AI system performed well in identifying screen-detected cancer cases, with results comparable to other studies using the same and other AI systems. Further, results concerning interval cancers and AI scores indicated a potential for the system to identify some of the cancers earlier than the radiologists. However, cancers with less suspicious features are challenging for AI and for radiologists, and how radiologists are influenced by the AI score and markings when interpreting mammograms is unclear. These aspects are important to bear in mind in the future implementation of AI in mammographic screening.

## Supplementary information

ELECTRONIC SUPPLEMENTARY MATERIAL

## Abbreviations

AI: Artificial intelligence
AUROC: Area under the receiver operating curve
CI: Confidence intervals
DCIS: Ductal carcinoma in situ
NT: Normal triaging
SN: Safety net
