# Supplementary material for: Retrospective evaluation of a CE-marked AI system, including 1,017,208 mammography screening examinations
Source: Eur Radiol. 2025 Mar 26;35(9):5685–94. doi: 10.1007/s00330-025-11521-4 (PMC12350444; doi:10.1007/s00330-025-11521-4)
Supplement: Supplementary file 1 — ELECTRONIC SUPPLEMENTARY MATERIAL [file 330_2025_11521_MOESM1_ESM.pdf]

Retrospective evaluation of a CE-marked AI system, including 1 017 208 mammography screening examinations

ELECTRONIC SUPPLEMENTARY MATERIAL

Supplementary Table 1. Number of examinations and area under the receiver operating curve (AUROC) + 95 confidence intervals (CI) by mammography equipment vendor estimated using the normal triaging score and safety net score by the artificial intelligence system. AUCROC was estimated only using screen-detected cancers (AUROC_SDC) as true reference status and AUCROC_IC+SDC were estimated using all cancers (interval cancers and screen-detected cancers) in the sample.

Mammography equipment	Number of examinations	Normal triaging score		Safety net score	
		AUROC_SDC (95% CI)	AUROC_SDC+IC (95% CI)	AUROC_SDC (95% CI)	AUROC_IC+SDC (95% CI)
Siemens (Breast center 1-7)	599 358	0.978 (0.976-0.980)	0.927 (0.923-0.932)	0.978 (0.976-0.980)	0.929 (0.924-934)
Philips (Breast center 8)	71 087	0.961 (0.952-0.969)	0.900 (0.884-0.916)	0.959 (0.951-0.968)	0.883 (0.870-0.895)
Hologic (Breast center 9+10)[a]	237 105	0.976 (0.973-0.980)	0.921 (0.914-0.929)	0.976 (0.973-0.980)	0.923 (0.915-0.930)
GE (Breast center 10) [a]	109 658	0.958 (0.950-0.965)	0.895 (0.882-0.907)	0.957 (0.949-0.963)	0.895 (0.882-0.907)

[a] Breast center 10 used GE before May 2010 (2005-2010) and Hologic after (2010-2021)

Supplementary Table 2. Interpretation score by each of the two radiologists (R1 and R2) for screen-detected and interval cancers in N70, unclassified and P3, where N70 is the 70% with the lowest normal triaging (NT) score by the artificial intelligence system, P3 is the 3% with highest safety net (SN) score, and unclassified are cases not classified as negative (N70) or positive (P3).

	Screen-detected cancers			Interval cancers		
Interpretation score 1-5 by R1 and R2	N70, n=107	Unclassified, n=999	P3, n=4871	N70, n=625	Unclassified, n=843	P3, n=344
1+1	-	-	-	576 (92.2%)	646 (76.6%)	226 (65.7%)
1+2	40 (37.4%)	272 (27.2%)	462 (9.5%)	41 (6.6%)	142 (16.8%)	60 (17.4%)
1+3/4/5	16 (15.0%)	155 (15.5%)	585 (12.0%)	1 (0.2%)	13 (1.5%)	14 (4.1%)
2+2	29 (27.1%)	238 (23.8%)	462 (9.5%)	6 (1.0%)	35 (4.2%)	24 (7.0%)
2+3/4/5	10 (9.4%)	191 (19.1%)	829 (17.0%)	1 (0.2%)	6 (0.7%)	9 (2.6%)
3/4/5+3/4/5	12 (11.2%)	143 (14.3%)	2533 (52.0%)	0 (-)	1 (0.1%)	11 (3.2%)
